# An evaluation of a hepatotoxicity risk induced by the microplastic polymethyl methacrylate (PMMA) using HepG2/THP-1 co-culture model

**DOI:** 10.1007/s11356-024-33086-3

**Published:** 2024-04-02

**Authors:** Tugce Boran, Ozge Sultan Zengin, Zehra Seker, Aysenur Gunaydin Akyildiz, Mehtap Kara, Ezgi Oztas, Gül Özhan

**Affiliations:** 1 Department of Pharmaceutical Toxicology, Faculty of Pharmacy, Istanbul University, Istanbul, Turkey; 2 Department of Pharmaceutical Toxicology, Faculty of Pharmacy, Istanbul University-Cerrahpaşa, Istanbul, Turkey; 3 Institute of Graduate Studies in Health Sciences, Istanbul University, Istanbul, Turkey; 4 Department of Pharmaceutical Toxicology, Faculty of Pharmacy, Bezmialem Vakif University, Istanbul, Turkey

**Keywords:** Polymethyl methacrylate, Microplastic, HepG2/THP-1 co-culture model, Hepatotoxicity, Inflammation, Oxidative stress

## Abstract

Inappropriate disposal of plastic wastes and their durability in nature cause uncontrolled accumulation of plastic in land/marine ecosystems, also causing destructive effects by bioaccumulating along the food chain. Microplastics may cause chronic inflammation in relation to their permanent structures, especially through oxidative stress and cytotoxic cellular damage, which could increase the risk of cancer development. The accumulation of microplastics in the liver is a major concern, and therefore, the identification of the mechanisms of their hepatotoxic effects is of great importance. Polymethyl methacrylate (PMMA) is a widely used thermoplastic. It has been determined that PMMA disrupts lipid metabolism in the liver in various aquatic organisms and causes reproductive and developmental toxicity. PMMA-induced hepatotoxic effects in humans have not yet been clarified. In our study, the toxic effects of PMMA (in the range of 3–10 μm) on the human liver were investigated using the HepG2/THP-1 macrophage co-culture model, which is a sensitive immune-mediated liver injury model. Cellular uptake of micro-sized PMMA in the cells was done by transmission electron microscopy. Determination of its effects on cell viability and inflammatory response, oxidative stress, along with gene and protein expression levels that play a role in the mechanism pathways underlying the effects were investigated. The results concluded that inflammation, oxidative stress, and disruptions in lipid metabolism should be the focus of attention as important underlying causes of PMMA-induced hepatotoxicity. Our study, which points out the potential adverse effects of microplastics on human health, supports the literature information on the subject.

**Supplementary Information:**

The online version contains supplementary material available at 10.1007/s11356-024-33086-3.

## Introduction

In the last few years, when it has been realized that microplastics bioaccumulate in living things and have serious harmful effects on human health, many researchers have focused on this issue and the literature is rapidly growing (Prata et al. 2020; Sun et al. 2020; Yin et al. 2021; Weber et al. 2022). Many products such as medicines, shampoos, detergents, facial cleansers, toothpaste, make-up materials, synthetic clothing, and car tyres contain microplastics (Paco et al. 2017; Sun et al. 2020). Plastic waste is broken down into smaller sizes by mechanical or chemical processes such as hydrolysis and UV radiation, forming microplastics (diameter < 5 mm) and nanoplastics (diameter < 1000 nm) (Law and Thompson 2014; Hu and Palic 2020). As the size of plastics decreases, they become easier to take up by many organisms, however, more difficult to remove from the environment. Long-term exposure to these substances in the environment increases the risk of exposure to these plastics by entering the food chain, posing a threat to human health and ecological safety (Hu and Palic 2020). It is extremely important to elucidate the toxic effects and mechanisms of different microplastics on different systems due to the diversity, production, use, and therefore exposure sources of microplastics (Fadare et al. 2020; Sun et al. 2020; Yin et al. 2021). In 2022, global plastic production amounted to 400.3 million metric tons with an annual increase of 2.4%. It is estimated that at least 14 million tons of plastic waste end into the world’s oceans each year (https://bitly.ws/3fQqm). This rapid accumulation of plastic, produced from non-degradable polymers (Burelo et al. 2023), in nature raises concerns for the environment and human health. Moreover, analytical methods cannot be enough to determine microplastics in both human samples and the environment (Barbosa et al. 2020). Therefore, microplastic contamination may be more than as thought.

The abundance of microplastics in the environment makes them inevitable for humans to be exposed to microplastics. It is possible to be exposed to microplastics through inhalation as well as oral exposure. Microplastics are reported to mix easily with air, and their proportion in the air is higher in crowded areas as they are lighter than regular plastics. In addition, the use of products such as personal care products and cleaning materials containing microplastics could be considered as a source of dermal exposure. It is well known that there is a high potential for microplastics to absorb toxic chemicals (e.g., polycyclic aromatic hydrocarbons, organochlorine compounds), resulting in the spread of these chemicals throughout the food chain, posing a great risk to all living organisms (Kosuth et al. 2018; Prata et al. 2020; Atugoda et al. 2021). Consequently, all this means that humans are chronically exposed to microplastics and highly vulnerable to their harmful effects (Prata et al. 2020).

Microplastics accumulate more in the liver, while nanoplastics with smaller particles accumulate in the gut, brain, kidney, and gonads (Yin et al. 2021; Liu et al. 2024; Xu et al. 2024). Due to their high surface area, microplastics can cause oxidative damage, cytotoxicity, and translocate to other tissues, while their persistent structure may limit their clearance from the organism, leading to chronic inflammation that increases the risk of developing cancer. Furthermore, there may be a link between exposure to microplastics and the increasing incidence of immunological system and neurological disorders (Prata et al. 2020).

Polymethyl methacrylate (PMMA), also known as acrylic, acrylic glass, or plexiglas, is a transparent thermoplastic that is often used in sheet form as an alternative to glass due to its strength, lightweight, and resistance to breakage. It is used as a raw material in laboratory equipment on doors and windows; on motorcycle, car, and helicopter windscreens; on LCD screens and monitors; and in various household furniture such as tables, chairs, and lamps (Pawar 2016). It is also used in medical and health applications such as intraocular lenses, bone cement, and dental and dermal fillers. According to information submitted by the industry to the US Food and Drug Administration (FDA) under the Voluntary Cosmetic Registration Program (VCRP), PMMA is widely used in bath soaps, make-up, hair dyes, and personal cleaning/care products. It has been used in a wide range of cosmetic products (Becker et al. 2011; Pellegrino et al. 2023). Recently, PMMA microplastics have been detected in the human liver (Horvatits et al. 2022), cardiac tissue (Yang et al. 2023), and blood (Leslie et al. 2022). This can be interpreted as systemic exposure to PMMA.

The mechanisms of PMMA-induced hepatotoxic effects in humans have not yet been elucidated. In the present study, the toxic effects of PMMA on the human liver were investigated using the HepG2/THP-1 macrophage co-culture model, which is a sensitive immune-mediated liver injury model. This highly sensitive model was developed a few years ago in which HepG2 and differentiated THP-1 monocytes were co-cultured, where the differentiated THP-1 cells acquire macrophage characteristics and act like the Kuppfer cells, so the communication between the immune system and the liver is successfully simulated (Granitzny et al. 2017; Padberg et al. 2020; Wewering et al. 2017). Therefore, the HepG2/THP-1 co-culture model is more sensitive than monolayer cell models in detecting immune-mediated liver injury and provides a result closer to the in vivo response.

In our study, THP-1 monocyte cells were differentiated into THP-1 macrophage cells with phorbol 12-myristate 13-acetate (PMA) to establish the model. HepG2/THP-1 macrophage cells were cultured in the system separated by a porous membrane. As particle size is important for the distribution and accumulation of microplastics, PMMA with a particle size in the range of 3–10 μm was used in our study. The size and shape of the particle were examined and confirmed by scanning electron microscopy (SEM) analysis (Figure S1). The detection of cellular uptake of micro-sized PMMA was evaluated by transmission electron microscopy (TEM). The determination of cytotoxic effect and inflammatory response, determination of oxidative stress inducing potential, and gene and protein expression levels involved in the pathways underlying the effects were investigated.

## Materials and methods

### HepG2/THP-1 co-culture model

THP-1 (TIB-202, ATCC) cells and HepG2 (HB-8065, ATCC) cells were grown in RPMI 1640 medium (containing 10% fetal bovine serum and 1% penicillin/streptomycin/amphotericin). The cell culture medium was renewed every 2–3 days. THP-1 cells were not allowed to exceed 1 × 10^6^ cells/mL, and HepG2 cells were subcultured after they became 60% confluent.

The co-culture study was performed using 12 well plates with inserts (Nest, China). HepG2/THP-1 co-culture model was generated in our previous study (Boran et al. 2024). Briefly, THP-1 cells were seeded into the inserts 0.65 × 10^5^/cm^2^ and differentiated using phorbol 12-myristate 13-acetate (PMA). HepG2 cells were also seeded to 12-well plate at a density of 1.3 × 10^5^/cm^2^. Then, HepG2 and THP-macrophages were co-cultured (Figure S2), and both cells were exposed to PMMA in the range of 0.25–1 mg/mL, and the plate was incubated for 72 h.

### Cytotoxicity

Cytotoxicity was determined by 3-(4,5-dimethylthiazol-2-yl)-2,5-diphenyltetrazolium (MTT) in both mono- and co-culture. HepG2 and differentiated THP-1 cells were exposed to PMMA microparticles (3–10 μm) (Cospheric LLC, CA, USA) in monoculture for 72 h to create prolonged conditions. Of note, the cell viability of the 24 and 48-h exposure results was not significantly affected (Figure S3 and S4); therefore, the following experiments were conducted after 72-h exposure. In the determination of the cytotoxic effect in HepG2 cells in the co-culture model, THP-1 cells and the exposure medium were renewed after exposure. The MTT solution (5 mg/mL) was added to the HepG2 cells (well plate) and THP-1 macrophages (cell culture insert), and the cells were incubated at 37 °C for 3 h. The supernatant was removed, and DMSO was added to the wells to dissolve the formazan crystals. Changes in optical density were measured at 590 nm using a microplate plate reader (Biotek, Germany). Cell viability was calculated as a percentage of the control group.

### Determination of oxidative stress

#### Determination of reactive oxygen species (ROS) production

ROS production was measured using the 2′,7′-dichlorodihydrofluorescein diacetate (H_2_DCFDA) dye (Sigma Chemicals, USA) following 72 h of PMMA exposure in the range of 0.25–1 mg/mL. H_2_DCFDA dye solution (20 μM) was added after the cells have been trypsinized and washed, and the cells were then incubated at 37 °C. Using ACEA flow cytometry (Agilent, CA, USA), the fluorescence signal in the FITC channel (FL-1) was read after 30 min of incubation. With the aid of Novoexpress software (Agilent, CA, USA), the outcomes were examined. The results were expressed as a percentage of the median fluorescence intensity (MFI%), which was produced by multiplying the test sample’s MFI value by 100 and dividing it by the control sample’s MFI value.

#### Determination of oxidative stress markers

Oxidative DNA damage (8-hydroxy-2ʹ-deoxyguanosine (8-OHdG)) and lipid peroxidation (malondialdehyde (MDA)) markers were evaluated following PMMA exposure. The level of 8-OHdG (AFG Bioscience, USA) and MDA (Elabscience, China) was measured with a commercial ELISA kit according to the manufacturer’s guideline. Briefly, cells were dissociated with trypsinization, and cell suspension was collected and centrifuged. After that, cells were washed with PBS, and then, cell suspension was diluted with PBS. Cells were exposed to 3–5 times freeze–thaw process and ultrasonication for lysing cells and discharging intracellular components. After centrifugation, the supernatant was collected for ELISA assay procedure. For MDA ELISA procedure, 50 μL of bionylated detection antibody working solution was added to 50 μL sample, standard, and each dilution of blank. After incubation and washing processes, 100 μL HRP conjugate working solution was added and plate was washed for one time. After addition of 90 μL substrate reagent plate was incubated 15 min at 37 °C, the reaction was stopped with 50 μL stop solution. The optical density (OD) was determined using a microplate spectrophotometer (Epoch, Erlanger, Germany) at 450 nm. For 8-OHdG ELISA procedure, 40 μL sample diluent was added to 10 μL testing sample for the final fivefold sample dilution. The next steps for the assay procedure were similar with the MDA ELISA procedure. In addition, the protein level was determined by BCA assay (ThermoScientific, MA, USA). The results were expressed as ng/mL per mg protein and ng/L for MDA and 8-OHdG, respectively. The results were normalized to the control group after 72 h of treatment.

#### Determination of protein oxidation

OxyBlot protein oxidation detection kit (Sigma, USA) was used for the detection of protein oxidation. After 72 h of PMMA exposure, the cell lysates were prepared using RIPA lysis buffer (Santa Cruz Biotechnology, USA). The protein amounts were measured using Take3 Plate (Biotek, Germany). Two aliquots of each sample were processed according to the manufacturer’s instructions. One aliquot was subjected to the derivatization reaction while the other was prepared as a negative control. After the separation of protein samples by sodium dodecyl sulfate–polyacrylamide gel electrophoresis (SDS-PAGE) and transfer to PVDF membrane using the Trans-Blot Turbo System (Bio-Rad, USA), 1% BSA/PBS-T was added to the membrane to block non-specific sites and the membrane was incubated for 1 h. Following primary antibody and HRP conjugated secondary antibody applications, the chemiluminescent reagent (Thermo Scientific, USA) was applied to the membrane and protein bands were imaged by Fusion FX (Vilber, France). The results were analyzed densitometrically with the ImageJ software.

### Determination of antioxidant response

For the determination of oxidative stress response, the levels of GSH, and SOD2 and CAT enzyme activities were examined after 0.25–1 mg/mL PMMA exposure for 72 h. The changes in GSH were assessed using a commercial ELISA kit (Elabscience, China) according to the manufacturer’s protocol. The results were expressed as μg/mL per mg protein. The SOD2 and CAT activities were evaluated using commercial kits (Elabscience, China) according to the manufacturer’s protocol. The cells (1 × 10^6^/mL) were homogenized, and supernatant was used after centrifugation. For SOD2 and CAT activity determination, 20 μL of enzyme diluent was added to 20 μL of sample. Then, the substrate application solution (200 μL) was added to each well and incubated. After 20 min incubation at 37°C, absorbance was measured with a microplate reader (Epoch, Germany) at 450 nm. The antioxidant enzyme activities were calculated as U/per mg protein, and all results were normalized to the control group after 72 h of treatment.

### Determination of inflammatory response

After 72 h of PMMA exposure in the range of 0.25–1 mg/mL, the levels of TNF-α, IL-1β, IL-6, IL-8, IL-10, IL-12, IL-17, IL-18, IL-23, IL-33, MCP-1, INF-α, and IFN-γ were assessed by LEGENDPlex™ human inflammation panel (Biolegend, USA) using BD Accuri flow cytometry (BD Biosciences, USA) following the manufacturer’s protocol. The cell culture supernatant was used for the determination of cytokine secretion. The size of the beads is represented in the FL-3 channel, and the fluorescence intensity in the FL-2 channel correlates proportionally with cytokine concentrations (pg/mL). The cytokine concentration was calculated using the standard curve, and the results were normalized to the control group.

### Determination of NFκB protein expression

NFκB expression at protein level was determined with western blot analysis. After protein isolation using with RIPA lysis buffer system (Santa Cruz Biotechnology, USA), extracted proteins were separated by sodium dodecyl sulfate-polyacrylamide gel electrophoresis (SDS-PAGE) and then transferred to 0.22-μm PVDF membrane (BioRad, USA). Following membrane blocking with 5% non-fat dry milk, membrane was incubated with NFκB primary antibody (1:1000 dilution) (ab16502, Abcam, UK) for 1 h at room temperature. After washing steps, membrane was treated with HRP-conjugated secondary antibody (1:20,000 dilution) (ab97051, abcam, UK). Membranes were imaged using a chemiluminescence imaging system (ChemiDoc, Biorad, USA). β-Actin (1:1000 dilution) (sc-47778, Santa Cruz Biotechnology, USA) was used as housekeeping protein. Results were quantified using ImageJ software and normalized to the control group.

### Determination of gene expressions

The expressions of *HO-1* (heme oxygenase-1) for oxidative damage and *PPARα* (peroxisome proliferator-activated receptor alpha), *PPARγ (*peroxisome proliferator-activated receptor gamma), *FABP1* (fatty acid binding protein1), *LXR-α* (liver X receptor alpha), and LDLR (low-density lipoprotein receptor) genes for lipid metabolism were determined in HepG2 cells after 72 h of exposure to the range of 0.25–1 mM concentrations of PMMA in co-culture. The RNA isolation from cell cultures was performed using an RNA isolation kit (Roche, Germany) according to the manufacturer’s instructions. cDNA from isolated RNAs was synthesized by using a OneScript Plus cDNA Synthesis Kit (abmGood, Canada) following the manufacturer’s instructions. The expression analysis of the relevant genes was performed by qRT-PCR after optimization of primer design. β-Actin was used as a reference gene, and the expression of the target gene was measured by comparing it with the reference gene (target gene average Cp/reference gene average Cp), and any changes in gene expression were determined by comparing the control and exposure groups. The primer sequences and their annealing temperatures are given in Table 1.

**Table 1:** Primer sequences for the evaluation of gene expression level

Gene	Primer sequence (5′-3′)	Tm (°C)	Reference
*HO-1*	**F:** ATgACACCAAggACCAgAgC **R:** gTgTAAggACCCATCggAgA	60	Maeda et al. (2008)
*PPARα*	**F:** CATTACggAgTCCACgCgT **R:** ACCAgCTTgAgTCgAATCgTT	58	Rogue et al. (2011)
*PPAR-γ*	**F:** CTgAATgTgAAgCCCATTgAA **R:** gTggAAgAAgggAAATgTTgg	58	Harada et al. (2005)
*FABP1*	**F:** TgTCggAAATCgTgCAg **R:** gATTATgTCgCCgTTgAgTT	53	Wang et al. (2005)
*LXR-α*	**F:** AACCCACAgAgATCCgTCCACAAA **R:** ATTCATggCCCTggAgAACTCgAA	63	Sangkitikomol et al. 2010
*LDLR*	**F:** CAATgTCTCACCAAgCTCTg **R:** TCTgTCTCgAggggTAgCTg		Sangkitikomol et al. (2010)
*β-actin*	**F:** ACTACCTTCAACTCCAT **R:** TgATCTTgATCTTCATTgTg	48	Rosa et al. (2009)

### Determination of intracellular neutral lipid accumulation

The intracellular accumulation of neutral lipids was evaluated by light microscopic imaging with Oil Red O (lipid) staining kit (Sigma-Aldrich, USA). After the cells were exposed to PMMA for 72 h, they were first fixed with formalin (10%) according to the manufacturer’s instructions. After performing the required steps, hematoxylin was added to the cells and the cell nuclei were stained. The lipid droplets were visualized as red in the cells examined under the light microscope (Nikon, Eclipse-Ti-S). Quantification of lipid accumulation was performed via captured bright-field images using magnification of × 20 by using ImageJ software as previously described by Mehlem et al. (2013).

### Transmission electron microscopy (TEM) imaging

The cells were treated with PMMA (1 mg/mL). Following 72-h treatment, HepG2 cells were harvested and were fixed with glutaraldehyde solution (2.5%) at + 4 °C for 24 h. Afterward, the cells were washed with PBS (1 ×) five times before being fixed in OsO_4_ (1%) for 1 h. The cells were embedded in agar (4%). The agar block was dehydrated with varying concentrations of ethanol, and the agar block was embedded in resin. Ultra-thin sections were obtained with an ultra-microtome (Leica, Germany) and were analyzed with FE-SEM microscope in the STEM mode (Hitachi Regulus 8230, Japan).

### Statistical analysis

Statistical analysis was completed by GraphPad Prism (version 6). The statistical differences compared to the control group were assessed by one-way ANOVA followed by the Tukey test. The outcomes are presented as mean ± standard deviation (SD). All assays were completed in triple biological replicates. All results were normalized to the control group. *p < 0.05, **p < 0.01, ***p < 0.001, ****p < 0.0001 values are considered as statistically significant.

## Results

### Cytotoxicity of PMMA

**Fig. 1:**
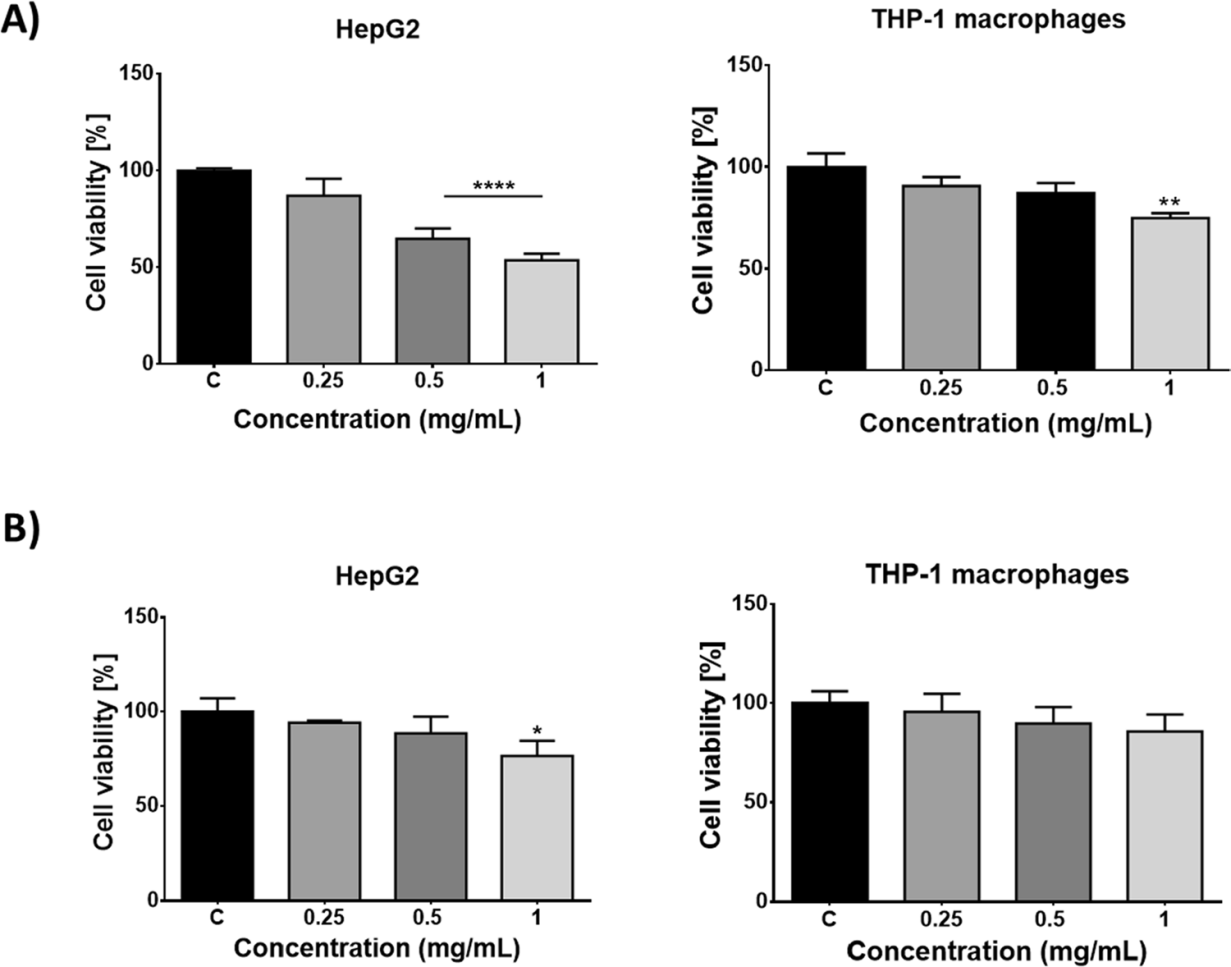
Effect of PMMA on cell viability in mono-cultured (**A**) and co-cultured (**B**) models. *p < 0.05, **p < 0.01 and ****p < 0.0001 vs control group

The MTT results indicate that PMMA significantly decreased cell viability in the monoculture HepG2 cells after 72-h exposure at 0.5 and 1 mg/mL concentrations (*p* < 0.0001) and in the THP-1 cells only in the 1 mg/mL concentration group (*p* < 0.01) (Fig. 1). In the co-culture model, the cell viability decreased significantly only in the HepG2 cells 1 mg/mL concentration group (*p* < 0.05) and was not significantly affected in the THP-1 cells in the concentration groups studied (*p* > 0.05).

### Determination of oxidative stress

**Fig. 2:**
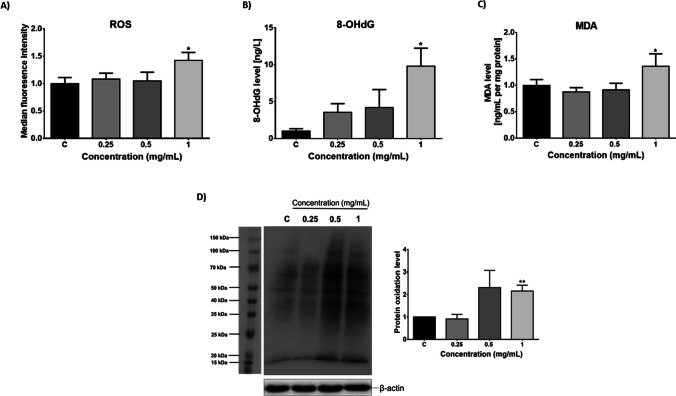
Changes in the levels of ROS (**A**), 8-OHdG (**B**), MDA (**C**), and oxyblot image and bar graph (**D**) induced PMMA exposure for 72 h. ROS, reactive oxygen species; MDA, malondialdehyde; 8-OHdG, 8-hydroxy-2ʹ-deoxyguanosine. *p < 0.05, **p < 0.01 vs control group

The levels of ROS and MDA were observed to increase significantly (*p* < 0.05) after 72 h of exposure to 1 mg/mL PMMA (Fig. 2). 8-OHdG levels showed a concentration dependent increase and were significantly upregulated in the highest concentration group (*p* < 0.05). Protein oxidation levels were also increased after 72-h exposure to 1 mg/mL PMMA.

**Fig. 3:**
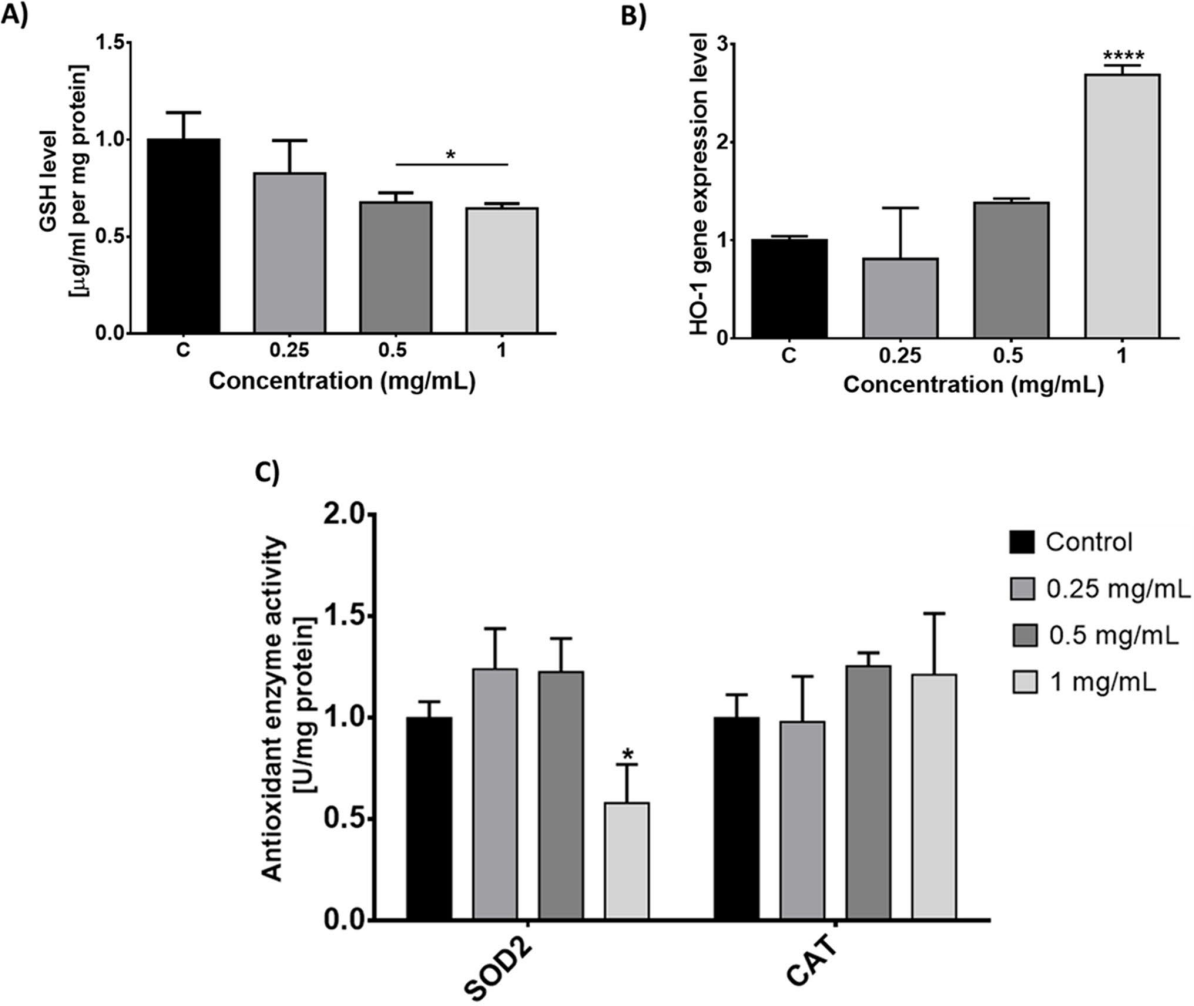
Changes in the levels of GSH (**A**), *HO-1* gene expression (**B**), and antioxidant enzyme activity (**C**) induced PMMA exposure for 72 h. GSH, reduced glutathione; SOD2, superoxide dismutase 2; CAT, catalase. *p < 0.05, ****p < 0.0001 vs control group

The levels of the antioxidant molecule GSH decreased in a concentration-dependent manner after PMMA exposure, and the increase was significant in the 0.5 and 1 mg/mL concentration groups (Fig. 3A). The expression of the *HO-1* gene, which plays a role in the antioxidant system, was upregulated in the higher concentration groups and downregulated in the lowest concentration group (Fig. 3B). A significant decrease in SOD2 activity (*p* < 0.05) was observed at the highest concentration of PMMA; however, an insignificant increase in activity was observed in the lower concentration groups (*p* > 0.05). CAT activity showed no significant change after PMMA exposure (Fig. 3C).

### Determination of inflammatory response

**Fig. 4:**
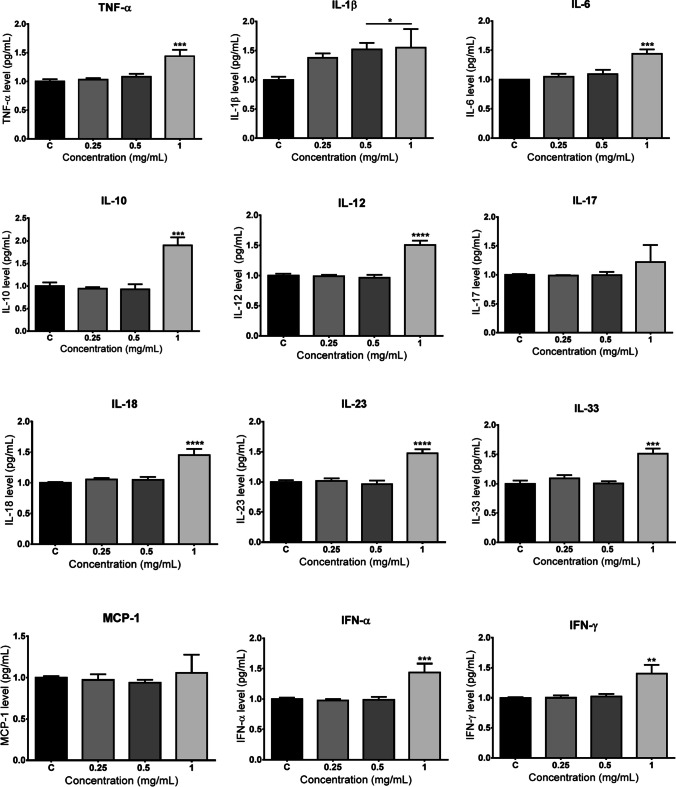
Changes on inflammatory mediator’s secretion following PMMA exposure for 72 h. IL, interleukin; MCP-1, monocyte chemoattractant protein-1; IFN, interferon. *p < 0.05, **p < 0.01, ***p < 0.001, ****p < 0.0001 vs control group

PMMA significantly induced secretion of TNF-α, IL-6, IL-10, IL-12, IL-18, IL-23, IL-33, IFN-α, and IFN-γ at 1 mg/mL concentration after 72-h exposure (*p* < 0.01), whereas increases in IL-17 and MCP-1 levels were not found to be statistically significant. An increase in IL-1β was observed after 72 h at 0.5 and 1 mg/mL PMMA (*p* < 0.05) (Fig. 4).

### Determination of NFκβ expression level

**Fig. 5:**
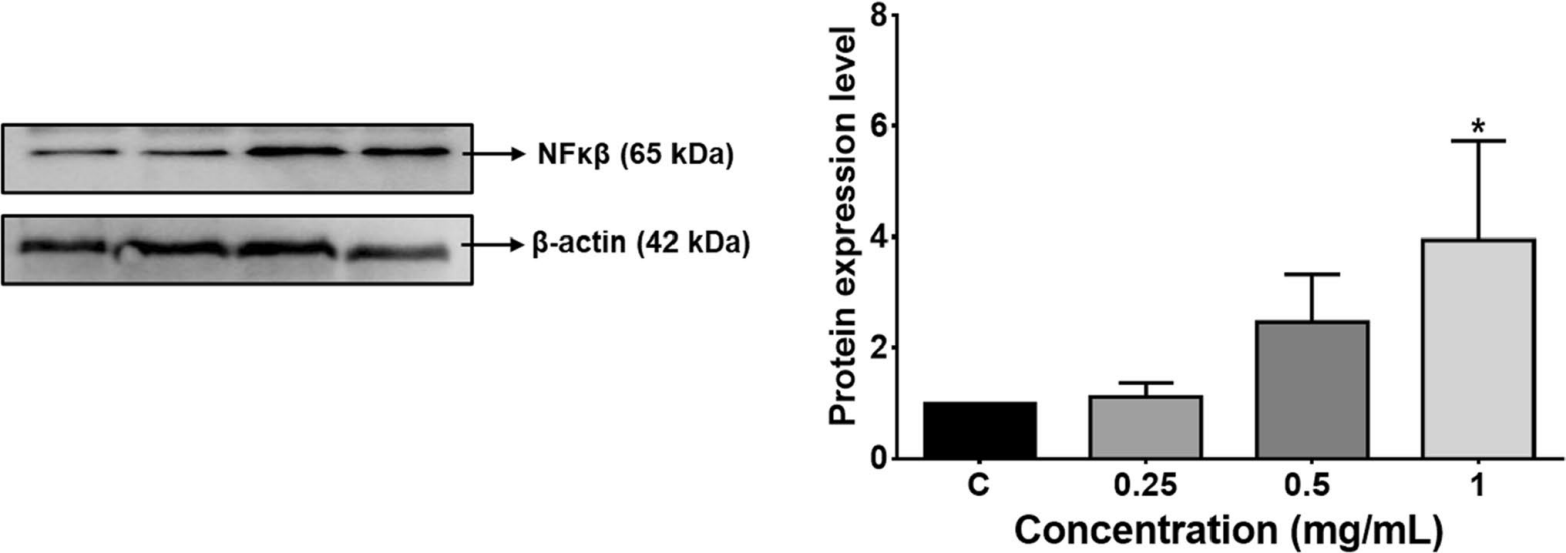
Changes in the expression of NFκβ at protein level. *p < 0.05 vs control group. NFκβ, nuclear factor kappa beta

NFκβ protein expression level was observed to enhance at 0.5 mg/mL and 1 mg/mL PMMA concentration, but this increase was found significant only following 1 mg/mL PMMA exposure (Fig. 5).

### Determination of expressions in lipid metabolism-related genes

**Fig. 6:**
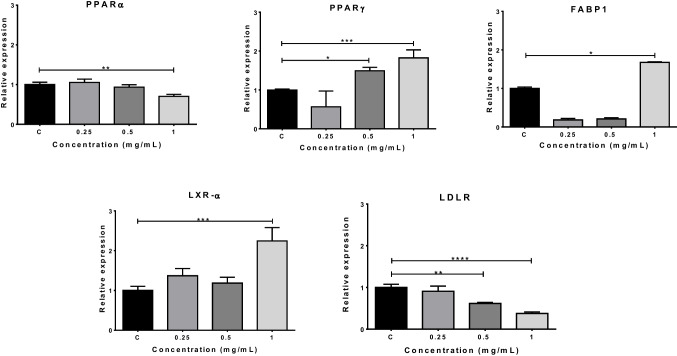
Changes in the levels of *PPARα*, *PPAR*γ, *FABP1*, *LXR-α*, and *LDLR* gene expressions following PMMA exposure for 72 h. *p < 0.05, **p < 0.01, ***p < 0.001., ****p < 0.0001 vs control group. PPARα, peroxisome proliferator activated receptor α; PPARγ, peroxisome proliferator activated receptor γ; FABP1, fatty acid binding protein 1; LXR-α, liver X receptor; LDLR, low-density lipoprotein receptor

The gene expression level of *PPARα* decreased after 1 mg/mL PMMA exposure (*p* < 0.01). *PPARγ* and LDLR expression were observed to be upregulated in the 0.5 mg/mL (*p* < 0.05) and 1 mg/mL (*p* < 0.001) concentration groups while an insignificant decrease was observed at the lowest concentration (Fig. 6). *FABP1*, however, showed a downregulation in the 0.25 and 0.5 mg/mL concentration groups and a significant upregulation in the 1 mg/mL group (*p* < 0.05). *LXR-α* expression showed significant increases at 1 mg/mL concentration (*p* < 0.001).

### Intracellular neutral lipid accumulation

**Fig. 7:**
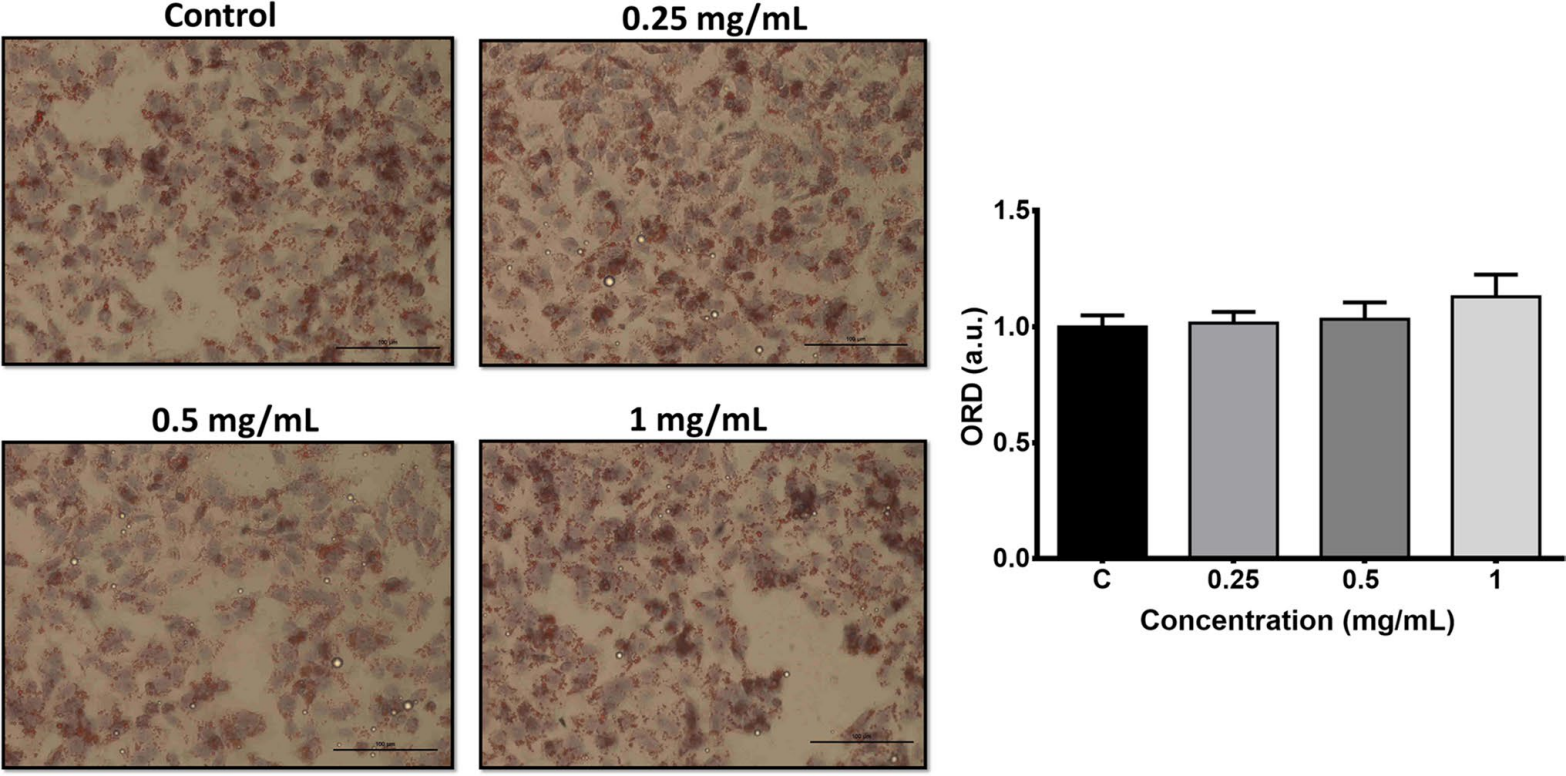
Representative light microscopic images after Oil Red O staining with hematoxylin showing the changes in intracellular neutral lipid accumulation following PMMA exposure for 72 h. Hemotoxylin (purple) indicates the nucleus of the cells, and Oil Red O (red) indicates neutral lipids. Images were captured using an × 20 objective. Bar graph represents the normalized density of lipid droplets in captured bright-field images quantified by using ImageJ software. ORD, oil red O density; a.u., arbitrary unit

As shown in Fig. 7, intracellular neutral lipid accumulation was slightly increased at the highest concentration after 72-h PMMA exposure (*p* > 0.05).

### TEM imaging results

**Fig. 8:**
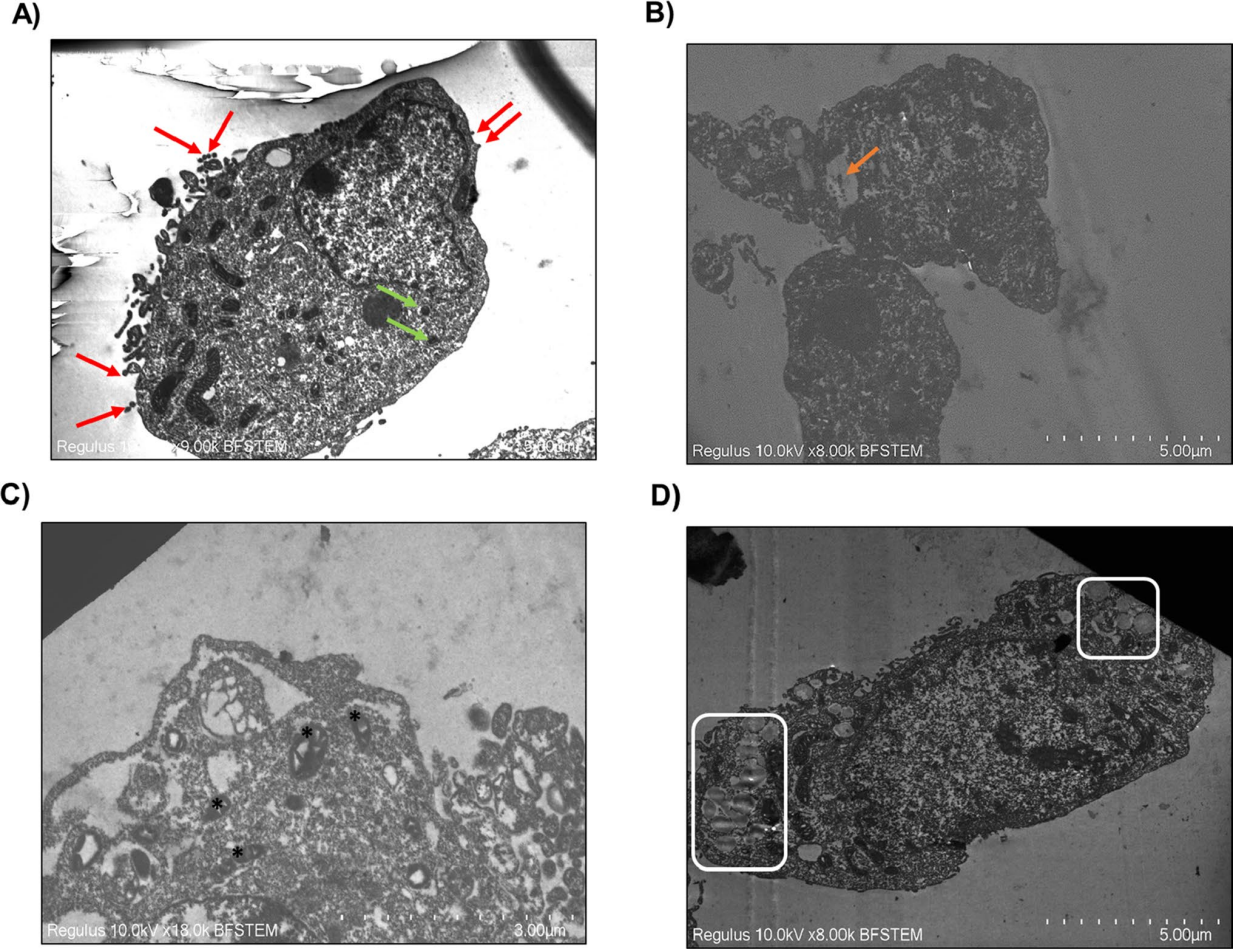
TEM imaging results following 1 mg/mL PMMA exposure for 72 h in HepG2 cells. Red arrows show microparticles attached to the membrane surface; green arrows show microparticles engulfed within the cell (**A**). Orange arrow shows microplastic-like structures enclosed in vacuoles (**B**). Asterisks indicate severely damaged mitochondria (**C**). White boxes show the lipid droplets (**D**)

While some of the microparticles were taken up into the cell, most adhered to the cell membrane as shown in Fig. 8. TEM analysis showed that PMMA induced mitochondrial damage and an increase in lipid droplets.

## Discussion

Microplastics can cause chronic inflammation, and this may increase the risk of cancer, particularly through oxidative stress and cytotoxic cell damage. Although microplastics have been found to accumulate in the liver, the mechanisms of PMMA-induced hepatotoxic effects in humans have not been elucidated.

In the present study, the toxic effects of PMMA on the human liver were investigated using the HepG2/THP-1 macrophage co-culture model which is a sensitive immune-mediated liver injury model. This highly sensitive model was developed several years ago in which HepG2 and THP-1 cells are co-cultured. In this model, the THP-1 cells act like the liver and the communication between the hepatocytes and the liver is successfully simulated (Granitzny et al. 2017; Padberg et al. 2020; Wewering et al. 2017). Therefore, the HepG2/THP-1 co-culture model is more sensitive than monolayer cell models in detecting immune-mediated liver injury and provides a result closer to the in vivo response.

PMMA was observed to cause a significant increase in ROS, which is an important cause of oxidative stress, along with the increase in MDA, which is indicative of lipid peroxidation in the present study. An important consequence of oxidative stress is DNA damage, and 8-OHdG is one of the biomarkers used for oxidative stress-induced DNA damage (Valavanidis et al. 2009; Gonzalez-Hunt et al. 2018). Therefore, a concentration-dependent increase in 8-OHdG was observed after PMMA treatment, indicating possible oxidative DNA damage. Similarly, in a study on dental pulp cells, PMMA resins induced oxidative stress, apoptosis, and DNA damage that could be reserved with *N*-acetylcysteine (Zhang et al. 2019). Proteins can be oxidized by ROS, which impairs cell function via loss or gain of protein function, protein aggregation, etc. (Celi and Gabai 2015). PMMA also increased protein oxidation.

Importantly, the antioxidant GSH was observed to decrease after PMMA exposure, contributing to the state of the possible oxidative stress. On the other hand, the observed increase in antioxidant enzyme activities is a sign that the cells are trying to cope with the increase in oxidative stress. The increase in CAT can be seen as an early sign of the activation of the antioxidant pathway. Similarly, a recent study in zebrafish reported an increase in CAT after PMMA exposure (Manuel et al. 2022). The mitochondrial antioxidant enzyme SOD2 is important for cell protection and was shown to be increased in the lower PMMA concentration groups in the present study. The early activation of SOD2 may be due to an immediate response to oxidative stress; however, its activity seems to decrease in the highest concentration group, possibly due to its toxicity, which is indeed more severe. Namely, low levels of ROS induce the antioxidant enzymes whereas sublethal levels may inhibit the enzymes (Dhoke et al. 2018). The effect of PMMA on SOD2 activity can also be read as a biphasic dose–response, known as the hormesis effect. A recent study investigating the toxic effects of nanoplastics on plants explains this phenomenon showing that low concentrations of microplastics stimulate positive responses and higher concentrations cause inhibitory effects (Yildiztuga et al. 2022). According to the gene-level analysis, *HO-1* levels also show a similar pattern. HO-1 is involved in the Keap-1-Nrf2 antioxidant pathway, and an interesting recent study reveals the hepatotoxic effects of polystyrene microplastics, which also induced the activation of this pathway and was subsequently attenuated by hydrogen sulfide (Li et al. 2021).

Inflammation is one of the main causes of the hepatotoxicity induced by chemicals (Luster et al. 2001). Our study shows that PMMA caused an upregulation of all pro- and anti-inflammatory mediators studied. More specifically, the pro-inflammatory cytokines TNF-α and IL-6 were observed to increase in the highest concentration group of PMMA, and IL-1β increased in a concentration-dependent manner. These cytokines are involved in the regulation of inflammatory responses. A recent study on primary human monocytes and dendritic cells reports that nanoplastics affect the release of these cytokines along with an increase in the anti-inflammatory cytokine IL-10 (Weber et al. 2022). Similarly, in the present study, IL-10 increased twofold after exposure to 1 mg/mL PMMA. IL-10 is a cytokine that inhibits the expression of inflammatory cytokines such as TNF-α, IL-6, and IL-1 by activated macrophages, so it can be said that the upregulation of the inflammatory cytokines would have been even higher if IL-10 was not active. Furthermore, TNF-α and IL-18 also act as inhibitors of pro-inflammatory cytokines under various conditions (Zhang and An 2007). On the other hand, the results of our study show that IL-12, IL-17, IL-18, IL-23, and IL-33 were all upregulated in the highest concentration group of PMMA. Similar functions can be induced by different cytokines as they are produced in a cascade that activates the production of other cytokines (Zhang and An 2007). IL-18 is known to induce IFN-γ production in cooperation with IL-12 (Yoshimoto et al. 1998) which is reasonably upregulated in our study. In an interesting study investigating the effects of biomaterials containing PMMA and such surface chemicals, cultured THP-1 cells on these biomaterials observed an overall increase in cytokines and chemokines (Schutte et al. 2009). Chemokines are a subgroup of cytokines known to induce chemotaxis. Similarly, MCP-1, a chemokine, acting as a proinflammatory mediator, was observed to increase in the highest concentration group of PMMA in our study. NFκβ plays a crucial role in the development inflammatory response and induces proinflammatory cytokines and chemokines (Ahmadi et al. 2021). Increase in inflammatory mediator following 1 mg/mL PMMA exposure may be associated with the upregulation of NFκβ expression. Additionally, inflammation can be triggered by oxidative stress (Hussain et al. 2016). Inflammatory processes can also cause oxidative stress and diminish the cellular antioxidant capacity (Khansari et al. 2009). In the present study, the oxidative stress induced by PMMA exposure may contribute the inflammatory reactions or vice versa.

FABPs are crucial molecules that work in accordance with PPARs, LXRs, and other mediators including hepatocyte nuclear factors, and sterol regulatory element-binding proteins in order to the maintain lipid balance, membrane integrity, and inflammatory response in the cell (Makowski and Hotamisligil 2004). PPARα, PPARγ, FABP1, LXR-α, and LDLR are expressed in the liver (Van De Sluis et al. 2017; Schulman 2017; Mukai et al. 2017; Berthier et al. 2021). FABP1 plays a critical role in facilitating fatty acid uptake and intracellular transport. FABP1 silencing in the liver has been reported to cause decrease in steatosis, inflammation, and oxidative stress (Wang et al. 2015; Mukai et al. 2017). PPARγ also regulates lipogenesis through interaction with FABP1 and inflammation (Morán-Salvador et al. 2013). In our study, an insignificant downregulation of *FABP1* was observed at the lower concentrations of PMMA which can be thought of as a protective mechanism of the cell, but it was significantly upregulated at the highest concentration. This may be the main reason for the observed levels of inflammatory markers and increase in lipid droplets in TEM images. On the other hand, *PPARγ* did not show significant change 0.25 and 0.5 mg/mL, but it was observed to be upregulated in the higher concentration groups of PMMA. This upregulation is consistent with the upregulation of *FABP1* in the highest concentration of PMMA, and this increase may also promote the inflammatory process. PPARα is important for mitochondrial β-oxidation of fatty acids (Wang et al. 2020). In the study, PPARα was downregulated at the highest concentration which can be related to the mitochondrial damage after PMMA exposure. Additionally, a decrease in PPARα gene exhibiting protective properties against metabolic disorder and inflammation (Adamowicz et al. 2022) may potentiate PMMA-induced inflammation. The gene expression of LXR-α, also important gene for lipid homeostasis, increased at the highest PMMA dose. LDLR is responsible for maintaining plasma cholesterol levels and carries LDL to the liver (Van De Sluis et al. 2017). In the study, LDLR expression was downregulated after PMMA exposure. Similar to our results, nano-sized PMMA particles negatively affect the expression of genes involved in lipid metabolism in the liver of European sea bass (*Dicentrarchus labrax*) and have the potential to trigger immune responses (Brandts et al. 2018).

Furthermore, lipid droplets were observed to increase in TEM analysis after PMMA exposure, although the increase in neutral lipid content was not significant, which may be due to polar lipid accumulation, such as phospholipids, glycolipids, and sphingolipids. Lipid droplets play an essential role in the connection between organelles and contribute to the regulation of the oxidative status of cells (Olzmann and Carvalho 2019). Finally, according to the TEM analysis, most of the PMMA microplastics presented, between the size of 3–10 µm, adhered to the cell membrane, potentially causing mechanical damage, leading to oxidative stress and inflammation.

## Conclusion

As the liver is the organ with the accumulation of microplastics and nanoplastics, it is important to elucidate the underlying mechanism of hepatotoxicity caused by the widely used microplastic PMMA. This study reveals that PMMA induces inflammatory responses by upregulating inflammatory markers and causing oxidative stress which was shown with an increase in ROS, MDA level, and protein oxidation and a decrease in antioxidant capacity (GSH depletion, SOD2 activity depletion), probably associated with mechanical damage of the particles, leading to hepatotoxicity. Disruptions in lipid metabolism may play a role in PMMA-induced hepatotoxicity. We conclude that inflammation and oxidative stress should be focused on as important underlying causes of PMMA microplastic-induced hepatotoxicity. Additionally, further comprehensive in vivo studies are needed to be done for real-life exposure situations.

The strength of our study is that the co-culture model we used in our study can show liver damage and provide similar results to in vivo studies in terms of oxidative damage, lipid metabolism, and inflammation simulation. Additionally, this study showed that PPMA exposure plays a role in liver damage through oxidative stress-mediated lipid metabolism disruption and inflammation development.

### Supplementary Information

Below is the link to the electronic supplementary material.

Supplementary file1 (DOCX 1568 KB)

## Data Availability

Data are available from the corresponding author upon reasonable request.
