# Supplementary material for: An evaluation of a hepatotoxicity risk induced by the microplastic polymethyl methacrylate (PMMA) using HepG2/THP-1 co-culture model
Source: Environ Sci Pollut Res Int. 2024 Apr 2;31(20):28890–904. doi: 10.1007/s11356-024-33086-3 (PMC11058773; doi:10.1007/s11356-024-33086-3)
Supplement: Supplementary file 1 — Supplementary file1 (DOCX 1568 KB) [file 11356_2024_33086_MOESM1_ESM.docx]

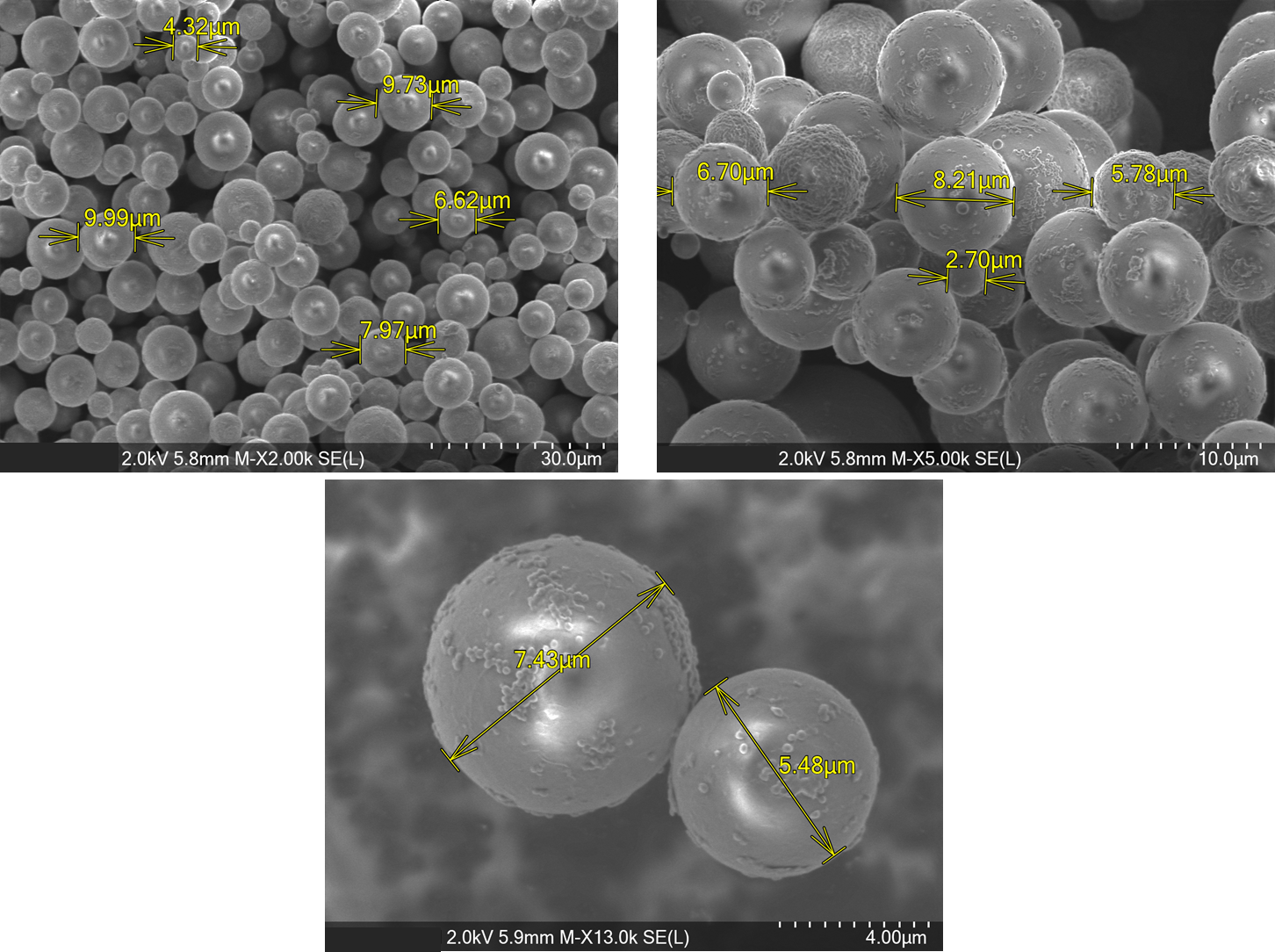


**Figure S1**: Scanning electron microscopy analysis results of PMMA microplastic. The size of the PMMA particles is 3-10 μm.


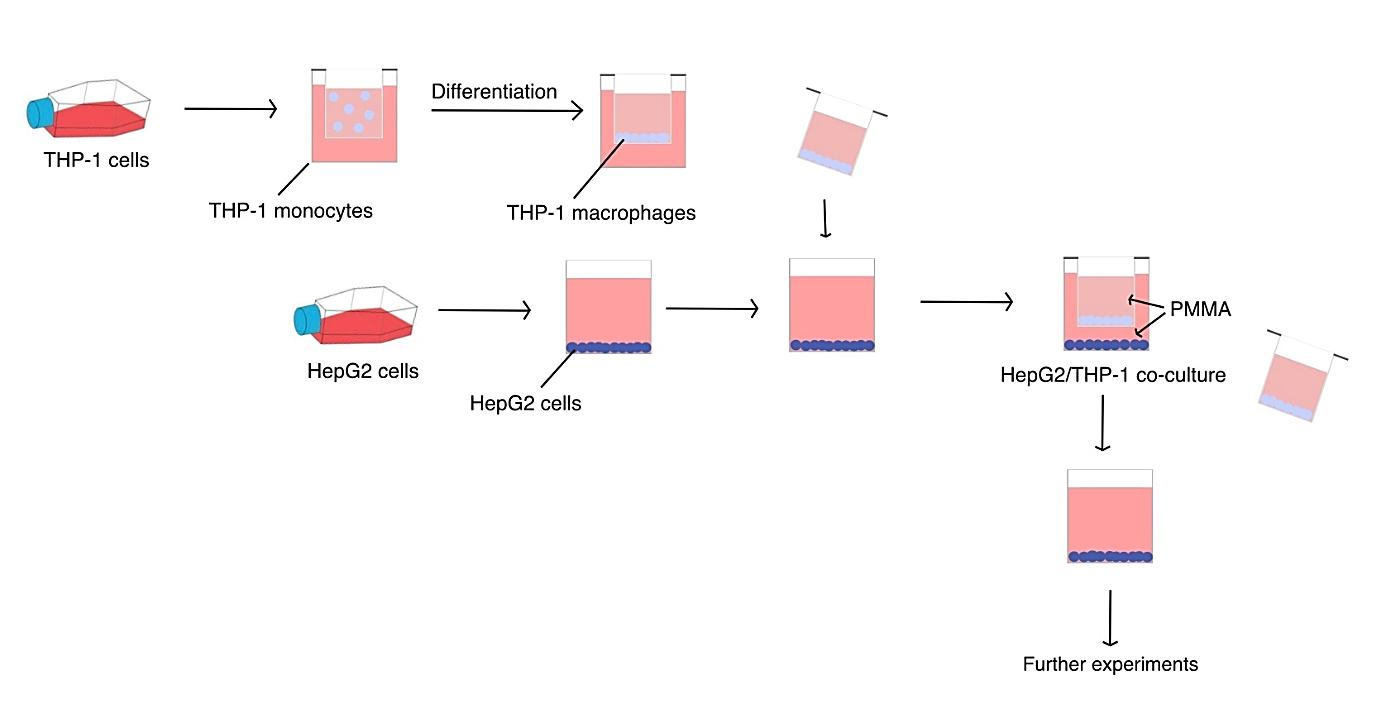


**Figure S2:** Schematic diagram of HepG2/THP-1 co-culture model.

**Figure S3**: Cytotoxic potential of PMMA microplastics in mono-cultured HepG2 cells and THP-1 macrophages following 24 h, 48 h, and 72 h exposure. *p<0.05, **p<0.01, ***p<0.001, ****p<0.0001 vs control group.

**Figure S4:** Cytotoxic potential of PMMA microplastics in co-cultured HepG2 cells following 24 h, 48 h, and 72 h exposure. *p<0.05 vs control group.
